# Fungal Peritoneal Dialysis-Associated Peritonitis as a Silent Threat: The Critical Role of Early Diagnosis and Catheter Removal

**DOI:** 10.7759/cureus.76191

**Published:** 2024-12-22

**Authors:** Mariana Freitas, Viviane Calice Silva

**Affiliations:** 1 Nephrology, Centro Hospitalar de Trás-os-Montes e Alto Douro, Vila Real, PRT; 2 Nephrology, Hospital Regional Hans Dieter Schmidt, Joinville, BRA

**Keywords:** chronic kidney disease (ckd), fungal infections, peritoneal dialysis catheter-associated peritonitis, peritoneal dialysis (pd), peritoneal dialysis peritonitis

## Abstract

Fungal peritonitis is an uncommon but serious complication that can occur in patients undergoing peritoneal dialysis. It represents a small percentage of all peritonitis cases in these patients. Its diagnosis can be challenging due to the slow growth of fungi and frequent negative culture results.

We report a case of fungal peritonitis in a 71-year-old man on automated peritoneal dialysis (APD). The patient presented with relapsing peritonitis unresponsive to antibiotics. During catheter removal, fungal hyphae were identified, confirming fungal peritonitis despite negative cultures. Prompt catheter removal and antifungal therapy with fluconazole led to infection resolution. The patient transitioned to hemodialysis.

This case highlights the importance of early suspicion and intervention in fungal peritonitis. Prolonged antibiotics and recurrent infections should raise concern for fungal etiology, particularly in refractory cases. Early catheter removal, combined with targeted antifungal therapy, is critical to successful outcomes. Our findings emphasize the diagnostic challenges of fungal peritonitis and the need for advanced microbiological techniques and collaborative care between clinicians and microbiologists.

## Introduction

Peritonitis is a serious and potentially life-threatening complication in patients undergoing peritoneal dialysis (PD). This infectious complication can compromise the function of the peritoneal membrane, leading to the loss of PD as a viable treatment option [1,2]. The most commonly identified pathogens in PD-associated peritonitis are bacterial agents, particularly gram-positive organisms (e.g., *Staphylococcus* species) and gram-negative bacilli, reflecting contamination or translocation processes [3]. Fungal peritonitis, although less common, is a feared complication due to its often severe clinical course and high morbidity and mortality rates [1,4,5]. The prevalence of fungal peritonitis among PD-related peritonitis cases varies globally but remains relatively low, ranging from 1% to 15% [1,4]. *Candida* species are the most commonly isolated fungal pathogens [1,6], but other opportunistic fungi such as *Aspergillus* and *Neosartorya* have been reported [7]. Risk factors for fungal peritonitis include prior antibiotic use for bacterial peritonitis and recent episodes of bacterial infection [8]. Its clinical presentation may have an insidious onset or resemble that of bacterial peritonitis, making early diagnosis challenging [6]. The clinical management of fungal peritonitis emphasizes prompt and aggressive intervention, including antifungal therapy and catheter removal. In fact, timely catheter removal is a cornerstone of management and is critical for reducing mortality and preventing further complications. Delayed recognition or treatment can lead to systemic fungal infection, organ failure, or death. This highlights the importance of maintaining a high index of suspicion for fungal etiology in cases of refractory or atypical peritonitis [1,7]. This case report aims to describe a patient with fungal peritonitis associated with automated peritoneal dialysis (APD) and to highlight the importance of considering fungal etiology as a differential diagnosis in PD-associated peritonitis, especially in refractory or supposedly relapsing cases. This report also pretends to emphasize the critical role of early catheter removal to improve the patient's prognosis.

## Case presentation

We report the case of a 71-year-old man with a medical history of arterial hypertension, type 2 diabetes mellitus diagnosed at the age of 37, ischemic heart disease with cardiac revascularization at age 55, and chronic kidney disease (CKD) in the context of diabetic kidney disease. In 2017, due to the progression of CKD to stage 5 and the patient's preference for hemodialysis, he was referred for the creation of an autologous vascular access. A left brachiocephalic arteriovenous fistula was constructed in October 2017, but a primary failure occurred. In January 2018, a right radiocephalic fistula was attempted but failed due to thrombosis. Given the poor vascular autologous access options, the patient was evaluated and considered a suitable candidate for PD. The patient's surgical history included only an appendectomy. In April 2018, a Tenckhoff catheter was placed by a surgeon using a laparoscopic approach, and urgent PD with low volumes was initiated during hospitalization due to the need for dialysis initiation. There were no complications in the immediate postoperative period, and the patient was discharged following clinical stabilization, maintaining APD at home. It is important to note that the patient maintained significant residual diuresis of 1.5 L/day.

Over the subsequent months, the patient demonstrated adequate metabolic control, although adjustments to the PD prescription were required to manage fluid balance, given his difficulty in adhering to fluid restriction despite preserved diuresis. The patient remained on PD for three years and eight months without significant complications, including infections.

**Figure 1:**
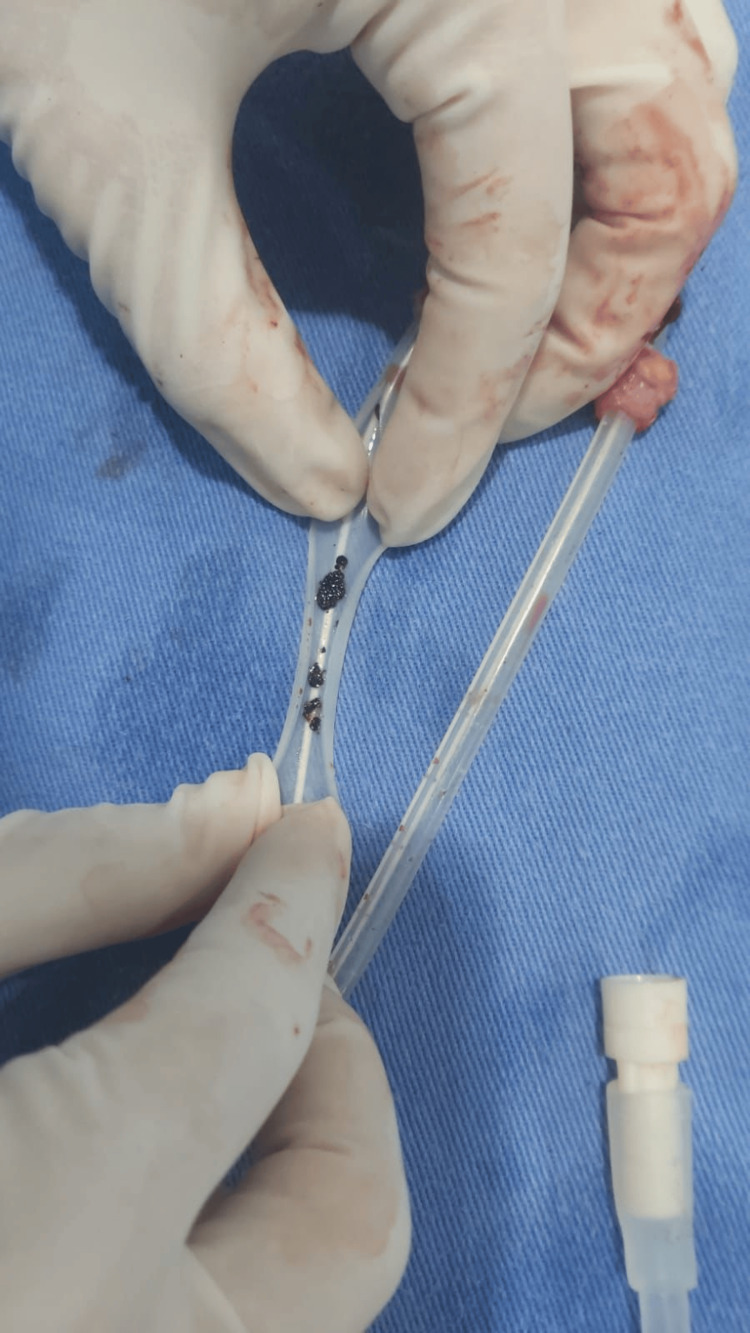
Photographic record of the removed peritoneal dialysis catheter showing fungal colonies.

In February 2022, the patient recurred to the PD unit for abdominal pain and cloudy dialysis effluent. Cytology of the peritoneal effluent confirmed peritonitis (leukocytes >100/µL, with >50% polymorphonuclear leukocytes), and empirical antibiotic therapy with intraperitoneal ceftazidime and vancomycin was promptly initiated, along with fluconazole prophylaxis. Cultures of the peritoneal effluent were negative. After three days of treatment, the patient showed clinical improvement with the resolution of symptoms and a reduction in effluent leukocyte count to <100/µL. A 14-day course of antibiotics was completed. One week after finishing antibiotic therapy, the patient developed relapsing peritonitis, presenting with abdominal pain, cloudy peritoneal effluent, and reduced peritoneal ultrafiltration. Cytological analysis of the peritoneal fluid again confirmed peritonitis (leukocyte count >100/µL with a predominance of polymorphonuclear cells), and empirical intraperitoneal ceftazidime and vancomycin were reinitiated. There was no concomitant infection of the exit site of the PD catheter. Dark spots suspected to be fungal colonies were observed on the catheter (Figure 1).

**Figure 2:**
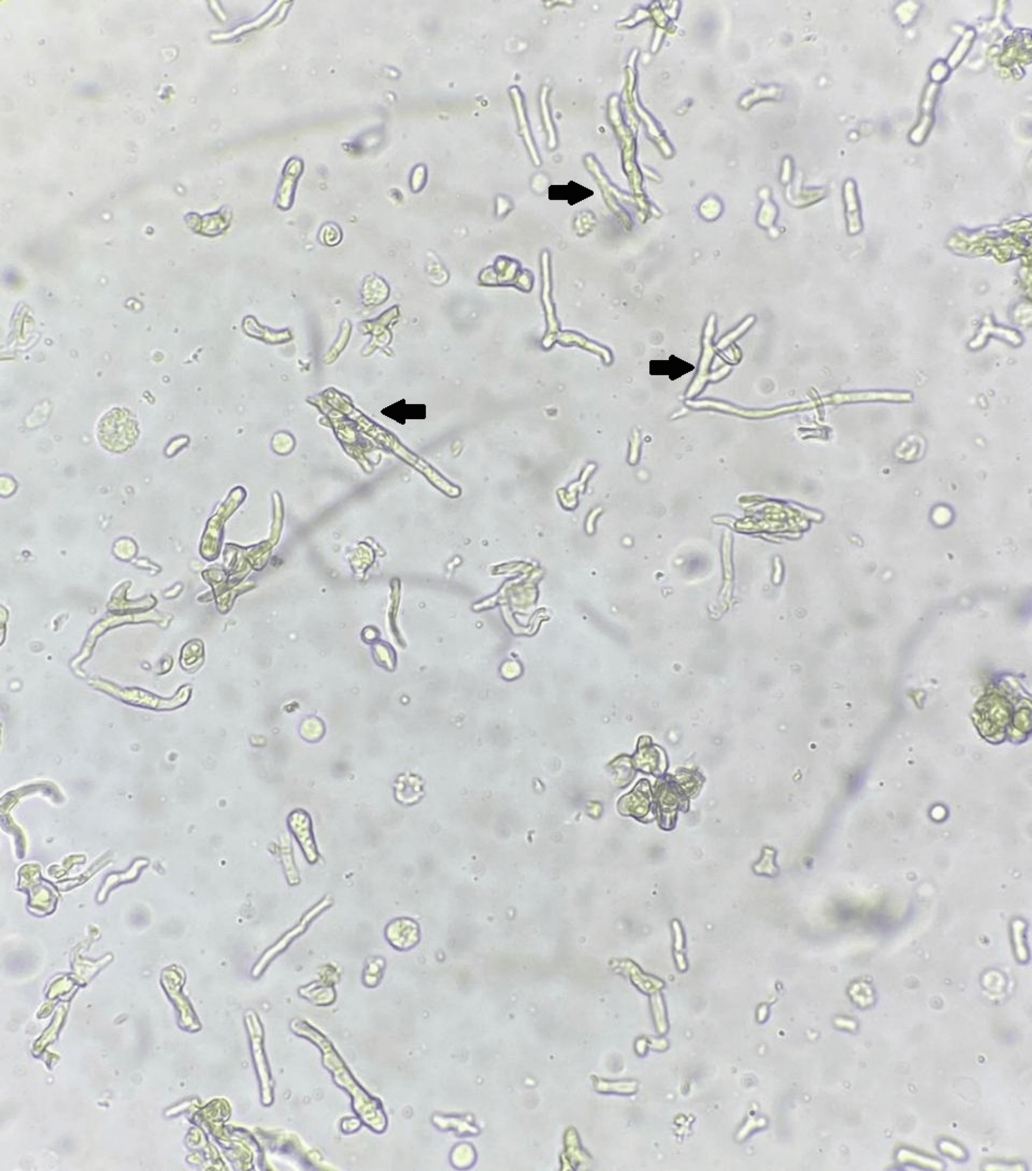
Image of the hyphae observed through a high-magnification lens. Image observed under a high-magnification lens, showing septated fungal hyphae (black arrows), characterized by transverse divisions (septa) along their filamentous structures.

Despite cytological findings showing a rapid decline in leukocyte counts, the etiological investigation remained inconclusive, with no microbiological isolates in the bacteriological (aerobic and anaerobic) and fungal cultures of the peritoneal effluent that were conducted. Also, acid-fast bacilli staining was negative. The patient was hospitalized for catheter removal due to relapsing peritonitis and the dark spots on the catheter. The observation of dark spots on the peritoneal catheter, combined with the identification of septated hyphae under a magnifying lens after catheter removal (Figure 2), was highly suggestive of fungal infection, even in the absence of culture confirmation. Thus, a diagnosis of fungal peritonitis was made. 

Antibiotic therapy was continued due to the possibility of an initial bacterial cause, and antifungal treatment with fluconazole was initiated (200 mg of loading dose, followed by maintenance dose with 100 mg). The patient showed a positive response to treatment, achieving resolution of the infection, and completed a 14-day course of combined antibiotic and antifungal therapy. It was not possible to repeat cytological or cultural analysis of the peritoneal effluent, considering that the catheter had been removed. Therefore, the resolution of the infection was assessed based on the absence of clinical symptoms and the absence of complications in imaging studies conducted after treatment. Since PD was no longer a viable option, the patient was switched to hemodialysis, initially using a temporary central venous catheter. This was subsequently replaced with a tunneled catheter placed in the right internal jugular vein. Upon discharge, the patient was enrolled in a chronic hemodialysis program.

## Discussion

Peritonitis remains one of the most serious complications of PD, carrying significant risks for the patient's survival and for the longevity of the peritoneal membrane. Mechanisms contributing to these outcomes include inflammation-induced fibrosis and vascular damage. Chronic inflammation resulting from peritonitis promotes angiogenesis, mesothelial injury, and sub-mesothelial fibrosis, which can culminate in impaired solute and fluid transport [9]. Furthermore, chronic exposure to dialysis solutions may exacerbate these changes, underlining the importance of prompt and effective treatment strategies [10].

Although bacterial pathogens, particularly gram-positive organisms such as *Staphylococcus epidermidis*, account for the majority of PD-related peritonitis cases, fungal peritonitis is a less common but more severe condition. In fact, this condition is linked to notably higher mortality rates than bacterial peritonitis, with studies indicating that mortality ranges from 20% to 30% [11,12]. Several factors can justify the greater severity of fungal peritonitis. Fungi, such as *Candida* species and *Aspergillus *species, exhibit virulence mechanisms, including biofilm formation on peritoneal catheters, which protect them from immune defenses and antifungal treatments, making eradication particularly challenging [11]. Moreover, they can provoke a more aggressive inflammatory response in the peritoneum and are opportunistic pathogens that prosper in immunocompromised conditions, commonly seen in dialysis patients [12,13]. Also, it is important to note that the diagnosis of fungal peritonitis is often delayed due to the slower growth of fungi in culture compared to bacteria, resulting in significant delays in initiating appropriate treatment. Lastly, in many cases, cultures yield negative results, complicating the identification of the causative agent and the selection of effective antifungal therapy [13].

Prolonged antibiotic use and recurrent episodes of bacterial peritonitis are well-known risk factors for fungal peritonitis [5,8]. In fact, most cases of fungal peritonitis occur following prior antibiotic treatment [5], and some studies indicate that 2-10% of PD patients develop fungal peritonitis following treatment for bacterial peritonitis [14].

Thus, fungal peritonitis must be considered in cases of refractory peritonitis, prolonged antibiotic therapy, or relapsing symptoms, as these factors are predisposing risks for fungal infections [1,15]. The most frequently implicated organisms are *Candida* species, including *Candida albicans*, *Candida glabrata*, and *Candida parapsilosis*. Other reported fungi include *Aspergillus*, *Trichosporon*, *Fusarium* species, and *Neosartorya*, albeit less frequently [6,7]. Identification of these organisms can be challenging due to the limited sensitivity of routine cultures, often requiring prolonged culture times or specialized microbial culture media (e.g., Sabouraud agar) [13,16]. Another factor that can justify negative fungal cultures in fungal peritonitis is the prior empirical antifungal therapy, which may inhibit fungal growth and decrease the sensitivity of culture methods. Additionally, biofilm formation by fungal organisms, such as *Candida* species, on PD catheters poses a significant challenge to culture due to their protective matrix [11]. In our clinical case, the absence of culture growth illustrates the diagnostic challenges associated with fungal peritonitis. We believe that the lack of fungal growth in cultures was due to prior antifungal prophylaxis, which may have suppressed fungal growth, and to the biofilm observed on the PD catheter, which could have protected the fungus within its matrix. Our experience with the reported case emphasizes that it is crucial to employ extended culture times, alternative media, and advanced microbiological techniques such as molecular diagnostic methods to detect less common agents, as already highlighted by previous authors [17,18]. It is also necessary to have a high clinical suspicion, taking into account the risk factors for fungal infections. In the present case, prolonged antibiotic use and the broad-spectrum therapeutic approach may have inadvertently favored fungal growth, even with prophylactic antifungal use. This aligns with evidence suggesting that antibiotics disrupt normal microbial flora, creating an environment favorable to fungal proliferation [3], as seen in the biofilm formed in the catheter. Preventive strategies for biofilm formation include stringent catheter care, regular monitoring of the exit site, and antifungal prophylaxis in high-risk patients, although its routine use must be balanced against the risk of antifungal resistance [5]. Furthermore, innovative approaches, such as antifungal catheter coatings and biofilm-disrupting agents, merit further research to enhance infection prevention, as the previously mentioned measures can sometimes fail, as observed in the patient from our case report.

The cornerstone of managing fungal peritonitis is the rapid removal of the PD catheter to eliminate the infectious focus, coupled with targeted antifungal therapy in an appropriate dose. Catheter removal is essential to eliminate the fungal biofilm, a primary source of persistent infection. Studies have consistently demonstrated improved outcomes with early catheter removal, even when cultures are negative, as this interrupts the infectious cycle and allows for effective antifungal therapy [19]. Delayed catheter removal is associated with higher mortality rates and an increased likelihood of transitioning to hemodialysis [20]. Furthermore, antifungal therapy should be initiated immediately, with agents such as fluconazole being a common choice for *Candida* species, the most frequent pathogens in fungal peritonitis. For resistant organisms or more severe cases, liposomal amphotericin B or echinocandins, such as caspofungin, may be used. Prolonged therapy, often lasting several weeks, is essential to ensure complete eradication and prevent recurrence [5]. In our clinical case, the removal of the catheter was promptly scheduled due to the suspicion of fungal peritonitis, and the patient was transitioned to hemodialysis. Our patient responded well to treatment with fluconazole at a therapeutic dose. Although the fungal species was not identified, the positive response to fluconazole and the observed hyphal morphology strongly suggest *Candida albicans* as the likely causative agent. The prompt control of the infectious focus through catheter removal and the timely initiation of antifungal therapy were crucial in achieving a favorable outcome for our patient.

According to current data, less than 20% of patients return to PD following an episode of fungal peritonitis [5,12]. The definitive transition to hemodialysis can be justified by different reasons. Firstly, as previously mentioned, inflammation caused by fungal infections often results in fibrosis and loss of peritoneal membrane function. Secondly, the high morbidity associated with fungal peritonitis discourages the return to the technique, as well as the risk of recurrence. In our patient, the option of returning to PD was not considered due to his preference to continue on hemodialysis and the high risk of peritonitis recurrence.

## Conclusions

PD-associated fungal peritonitis is a serious condition requiring prompt recognition and management. Our case reinforces the critical importance of considering fungal peritonitis in PD patients with atypical presentations or recurrent infections. Early diagnosis, prompt catheter removal, and tailored antifungal therapy are essential for improving outcomes in these patients. The collaboration between clinicians and microbiologists is crucial to address the diagnostic and therapeutic challenges posed by fungal peritonitis. Finally, efforts to improve diagnostic methods and prophylactic strategies are essential. Future research should focus on developing rapid, sensitive diagnostic tools, such as molecular techniques for early pathogen identification, and exploring novel antifungal agents and biofilm-disrupting therapies. Moreover, research regarding the effectiveness and safety of antifungal prophylaxis in high-risk populations could offer important guidance for improving preventive measures.
